# Identification of prognostic biomarkers and development of a prediction model for prostate cancer

**DOI:** 10.3389/fimmu.2025.1709264

**Published:** 2026-01-05

**Authors:** Dake Chen, Wu Chen, Ruxian Ye, Linjin Li, Feilong Miao, Xianghui Kong, Weiqiang Ning, Jingyi Jia, Qiuli Chen, Peter Wang, Bowei Yin

**Affiliations:** 1 Department of Urology Surgery, Wenzhou Third Clinical Institute of Wenzhou Medical University, Wenzhou People’s Hospital, Wenzhou Maternal and Child Health Care Hospital, The Third Affiliated Hospital of Shanghai University, Wenzhou, Zhejiang, China; 2 Department of Medicine, Beijing Zhongwei Medical Research Center, Beijing, China

**Keywords:** PC3, stem cells, prostate cancer, learning machine, immunotherapy

## Abstract

**Background:**

Prostate cancer (PCa) is biologically heterogeneous, and its molecular underpinnings remain incompletely define. In this study, we sought to identify genes shared between PCa cells and stem-like subpopulations and to develop a prognostic model.

**Methods:**

RNA sequencing was performed on PC3 cells and side population stem-like cells (SPC). Primary prostate tumor data were obtained from GSE172301, and The Cancer Genome Atlas (TCGA) provided transcriptomes with clinical annotations. Differential expression, immune microenvironment and infiltration analyses were conducted. Single-cell spatiotemporal transcriptomics data were analyzed using Seurat and spatialLibs. To delineate the role of PLXNA4 in PCa cells, we performed CCK-8 viability assays, EdU incorporation assays, Annexin V–FITC/PI flow cytometry for apoptosis, and Matrigel-coated Transwell invasion assays.

**Results:**

We identified 562 upregulated and 671 downregulated genes in SPC. A total of nine genes emerged, including CPNE6, RASL10B, GCNT4, STAC2, RBPMS2, PADI3, PLXNA4, S100A14, and MMP9, as potential targets using the support vector machine (SVM) and LASSO methods, with MMP9 highly expressed in tumor cells. A three-gene prognostic signature (RASL10B, RBPMS2, ANGPTL3) stratified patients into risk groups. The high-risk group showed enrichment of Gene Ontology terms related to immune activation, antigen receptor signaling, and B-cell–mediated immunity. We also cataloged seven ubiquitin-related markers and putative ubiquitination sites. Functionally, PLXNA4 depletion reduced cell viability and proliferation, increased apoptosis, and suppressed invasion in PCa cells.

**Conclusions:**

We identified nine target genes and propose a three-gene prognostic model for outcome prediction in PCa. Our findings suggest that targeting PLXNA4 may offer new therapeutic opportunities for the treatment of PCa, including immunotherapy.

## 1 Introduction

Prostate cancer (PCa) remains a major public health challenge, and its management has evolved rapidly (1, 2). As understanding of the genomic landscape and biology of primary and metastatic PCa has grown, diagnosis, staging, and treatment have improved (3–5). Nevertheless, despite advances in surgery, radiotherapy, and chemotherapy, the molecular drivers of PCa progression remain incompletely defined (6–8). Increasing evidence supports a hierarchical cancer stem cell (CSC) model in PCa and other malignancies (9, 10). CSCs possess self-renewal and pluripotency, and initiates tumorigenesis (11). CSCs play a crucial role in tumor relapse, metastasis, and mortality (12–14). Various molecular markers used to identify PCa CSCs include tumor stem cell markers (CD44, CD133) (15, 16), prostate stem cell markers (such as CD166) (17), and progenitor cell markers (Nestin, Oct4) (18, 19). Additional factors such as ATP binding cassette subfamily G member 2 (ABCG2) and SRY-box transcription factor 2 (SOX2) are often overexpressed in PCa and correlate with CSC abundance and proliferation (20, 21). Detecting these markers enables CSC isolation and characterization, with implications for diagnosis and therapy in PCa.

Recent studies position CSCs as a therapeutic target in rapidly growing, highly metastatic cancers (9, 22). Diverse prostate epithelial stem-cell populations initiate and drive PCa progression, and foster therapy resistance via lineage plasticity (23). Yet CSCs in PCa remain incompletely characterized. Therefore, deeper characterization of CSCs in PCa could clarify disease biology and enable more effective therapies, ultimately improving patient outcomes. To achieve this goal, high-throughput sequencing technology, also known as next-generation sequencing (NGS), could offer a practical path. By rapidly and cost-effectively profiling DNA and RNA at scale, NGS provides comprehensive views of the genomic and transcriptomic programs that drive tumor initiation, plasticity, and progression. Applied to PCa CSCs via bulk, single-cell, or spatial transcriptomic approaches, NGS can resolve lineage hierarchies, and nominate biomarkers for risk stratification and targeted intervention. Thus, systematic NGS-based profiling of CSCs in PCa is poised to accelerate mechanism-guided, precision treatments.

Protein post-translational modifications (PTMs) are covalent, enzymatic alterations that occur during or after protein synthesis and can significantly modify protein properties and functions (24). Beyond histones, common PTMs encompass a wide range of modifications, including acetylation, lactylation, methylation, ubiquitination, phosphorylation, and SUMOylation, which shape key cancer phenotypes (25). Abnormalities in PTMs have been observed to impact vital cellular functions, leading to aberrant proliferation, migration, and invasion (26). PTMs possess diagnostic and prognostic value and represent promising therapeutic entry points for PCa and other malignancies (27). For example, toosendanin stimulates apoptosis, ferroptosis, and M1 macrophage polarization through USP39-mediated deubiquitination of polo-like kinase 1 (PLK1) in PCa cells (28). Apolipoprotein E drives primary resistance to androgen receptor (AR)-targeted therapy by fostering tripartite motif containing 25 (TRIM25)-mediated androgen receptor (AR) ubiquitination, and enhances immunotherapy efficacy in PCa (29). Furthermore, mechanical signaling promotes immune escape through USP8-dependent, ubiquitination-driven degradation of programmed death ligand 1 (PD-L1) and major histocompatibility complex class 1 (MHC-1) (30). Collectively, ubiquitination-centered studies in PCa are of great significance for deciphering tumorigenesis mechanisms and advancing new treatments.

In this study, we sought to identify genes shared between PC3 cells and their stem-like counterparts and to build a prognostic model based on these genes. We hypothesized that integrating gene-expression profiles with clinical information would reveal robust biomarkers and enable construction of a model that accurately stratifies patient risk. To this end, we prioritized the analysis and discovery of ubiquitin-related biomarkers given their central roles in tumor biology. A validated prognostic signature could inform clinical decision-making, guide surveillance and treatment selection, and support precision therapy. Moreover, uncovering novel prognostic biomarkers may facilitate the development of individualized therapeutic strategies for patients with PCa.

## 2 Methods

### 2.1 Data collection

Expression and clinical data were collected from three sources. First, bulk RNA-seq was generated from the PC3 and SPC by a commercial provider. Raw reads underwent standard quality control, normalization, and downstream processing prior to analysis. Second, primary PCa data were obtained from the spatial single-cell transcriptomic dataset GSE172301. Third, transcriptomic profiles and clinical annotations for patients with PCa were retrieved from TCGA (PRAD). Inclusion required availability of gene-expression data, clinical covariates, and follow-up information. PCa cases with missing or incomplete data were excluded.

### 2.2 Differential expression analysis of key genes

Differentially expressed genes (DEGs) were identified using the limma package (false discovery rate, FDR < 0.05, |log2 fold-change| ≥ 1). Functional enrichment of significant DEGs was performed with clusterProfiler for Gene Ontology (GO) terms and Kyoto Encyclopedia of Genes and Genomes (KEGG) pathways. Results were visualized using volcano plots and heatmaps generated in R.

### 2.3 Immune microenvironment and infiltration analysis

Tumor and normal tissues were analyzed using R packages “CIBERSORT” (https://cibersort.stanford.edu/) to deconvolve the relative fractions of immune cell types from bulk expression data, and “ESTIMATE” (https://bioinformatics.mdanderson.org/estimate/) to compute stromal, immune, and composite ESTIMATE scores. Differentially abundant immune cell types between high- and low-risk cohorts were identified from these outputs.

### 2.4 Analysis of immunological checkpoints

Expression of canonical immune checkpoint genes was investigated from the transcriptomic data and contrasted across risk groups and clinical strata. The Cancer Immunome Atlas (TCIA) R package (https://tcia.at/home) was used as a reference for immune features and to contextualize checkpoint patterns. The amounts of expression of immune checkpoints were calculated and the relationship between the immune checkpoint expression and clinical outcomes were examined using the package.

### 2.5 Identification of ubiquitination−related biomarkers genes

Ubiquitination-related genes (URGs) were retrieved from GeneCards (https://www.genecards.org/) by searching keyword “Ubiquitination” (31). The “Venn” packages were utilized to identify the overlap between URGs and candidate biomarker genes (BGs). TRRUST (version 2, http://www.grnpedia.org/trrust) was employed to explore transcriptional regulation (32). A protein-protein interaction (PPI) network was constructed by utilizing STRING (Version 12.0, https://cn.string-db.org/) (33). Gene identifiers were harmonized with “org.Hs.eg.db” package, and functional enrichment was performed using “enrichplot” package. Moreover, “ggplot2” package was employed for generating bar plots and bubble charts. For gene list annotation and pathway aggregation, Metascape (https://metascape.org/gp/index.html#/main/step1) was employed (34).

### 2.6 Predictive analytics for ubiquitination−related biomarkers genes

GeneMANIA (https://genemania.org/) was utilized to provide multi-layer interaction networks that encompass protein-protein interactions, gene co-localization, genetic interactions, and shared pathways (35). Drug-gene interaction database (DGIdb, https://dgidb.org/) integrates multiple databases and literature-reported drugs, offering information on known and potential drug-gene interactions (36). The obtained drug-gene interaction relationships were imported into Cytoscape (Version 3.9.1) for network visualization. The ubiquitin-proteasome pathway was predicted and analyzed for ubiquitin ligase-substrate interactions using UbiBrowser 2.0 (http://ubibrowser.bio-it.cn/ubibrowser_v3/) (37).

### 2.7 SVM- and LASSO-based gene selection

The overlapping genes were selected using Lasso and SVM algorithms, which were implemented using the “glmnet” and “e1071” R packages, respectively. In the Lasso algorithm, non-informative genes were excluded by shrinking the coefficients towards zero using a penalty parameter. The overlapping genes with non-zero coefficients were selected. In the SVM algorithm, the overlapping genes were selected based on their importance in determining the optimal hyperplane by a kernel function to transform the feature space.

### 2.8 Single-cell sequencing analysis

Single-cell spatiotemporal transcriptomic analyses were carried out with R 4.1.3. Seurat (version 4.0.3), spatialLibs (version 1.6.1), and SingleR (Version 1.4.1) were used as R tools. Using the SingleR package, which annotates cells based on their gene expression patterns in comparison to a reference dataset, cell labelling was carried out.

### 2.9 Cell culture and transfection

Human PCa cell lines PC3 and DU145 were purchased from the Shanghai Cell Bank (Shanghai, China). Cells were propagated in RPMI-1640 supplemented with 10% FBS (Gibco) and antibiotics (100 U/mL penicillin and 100 μg/mL streptomycin). Cultures were maintained at 37 °C in a humidified incubator with 5% CO_2_. Cell identity was verified using short tandem repeat (STR) analysis, and routine mycoplasma tests confirmed negative status.

### 2.10 Transfection

Short hairpin RNA (shRNA) targeting PLXNA4 and a non-targeting control (shNC) were synthesized by GenePharm (Shanghai, China). PC3 and DU145 cells were plated and transfected with shRNAs using Lipofectamine 3000 (Invitrogen, USA). Target sequences were: shPLXNA4-1: GCT CTT AAC CAT TGA CGA TAA; shPLXNA4-2: GCA GAT AAA TGA CCG CAT TAA. Transfection efficiency was assessed by RT-PCR and Western blotting.

### 2.11 RT-PCR analysis

Total RNA was extracted from transfected PCa cells using TRIzol reagent (Invitrogen, USA). Moreover, cDNA was synthesized from 1 µg RNA with a reverse transcription kit. Quantitative PCR was performed with SYBR Green Master Mix (Applied Biosystems, USA). Relative PLXNA4 expression was calculated by the 2^–ΔΔCt^ method, using GAPDH as the internal control (38). Primer sequences are listed: PLXNA4 sense 5’-TCG TGC GGA TTG AGC CAG AAT G-3’, antisense 5’-TGA TGT GCT CCT TCC CTC CAT G-3’; GAPDH: sense: 5′-ACC ACA GTC CAT GCC ATC AC-3′, antisense: 5′-TCC ACC ACC CTG TTG CTG TA-3′.

### 2.12 Western blotting analysis

Transfected PCa cells were lysed in RIPA buffer to extract proteins, and protein concentrations were measured by BCA assay. Equal protein amounts were separated by SDS-PAGE and transferred to PVDF membranes. Membranes were blocked with 5% non-fat milk for 1 h, incubated overnight with anti-PLXNA4 antibody (1:1000, ab127892, Abcam) or anti-GAPDH antibody (1:2500, ab8245, Abcam), then with HRP-conjugated secondary antibodies for 1 h. Protein bands were visualized by enhanced chemiluminescence (ECL) detection system (39).

### 2.13 CCK-8 and EdU assays

Transfected PCa cells were seeded in 96-well plates for 72 h. After incubation, CCK-8 solution was added to each well for 2 h, and absorbance was measured at 450 nm to evaluate viability (40). Cell growth was assessed with an EdU detection kit (Beyotime, China). Transfected cells were plated in 24-well plates and maintained for 72 h, then exposed to EdU working solution for 4 h, fixed with 4% paraformaldehyde for 30 min. Nuclei were counterstained with Hoechst 33342. Fluorescence images were captured under a microscope, and EdU-positive fractions were analyzed using ImageJ software.

### 2.14 Annexin V-FITC/PI apoptosis assay

Apoptotic cell death was evaluated with an Annexin V-FITC/PI detection kit. After 72 h post-transfection, PCa cells were collected, rinsed, and resuspended in 500 μL of 1× binding buffer. Subsequently, 5 μL of Annexin V-FITC and 10 μL of propidium iodide (PI) were added for 15 min. The proportion of apoptotic cells was determined by flow cytometry, and early/late apoptotic populations were quantified (41).

### 2.15 Transwell invasive assay

Cell invasion was assessed using Matrigel-coated Transwell chambers (Corning, USA). Medium containing with 10% FBS was placed in the lower chamber as a chemoattractant, while transfected PCa cells suspended in serum-free medium were seeded into the upper chamber. After 24 h, non-invading cells on the upper surface were removed, and cells that migrated to the underside were fixed with 4% paraformaldehyde, stained with crystal violet, and visualized. The number of invaded cells was quantified microscopically (42).

### 2.16 Statistical analysis

The statistical analyses were performed using R software (version 4.1.3). To compare continuous variables between high-risk and low-risk groups, the Student’s t-test and Wilcoxon rank-sum test were utilized, while the chi-square test was used to compare categorical variables. Survival probability was estimated by the Kaplan-Meier technique, and the log-rank test was performed to compare the survival curves of the two groups. Additionally, multivariate Cox regression analysis was conducted to evaluate the prognostic value of immune cells. Tests were two-sided with P < 0.05 considered statistically significant.

## 3 Results

### 3.1 Enrichment analysis of KEGG and GO pathways

**Figure 1:**
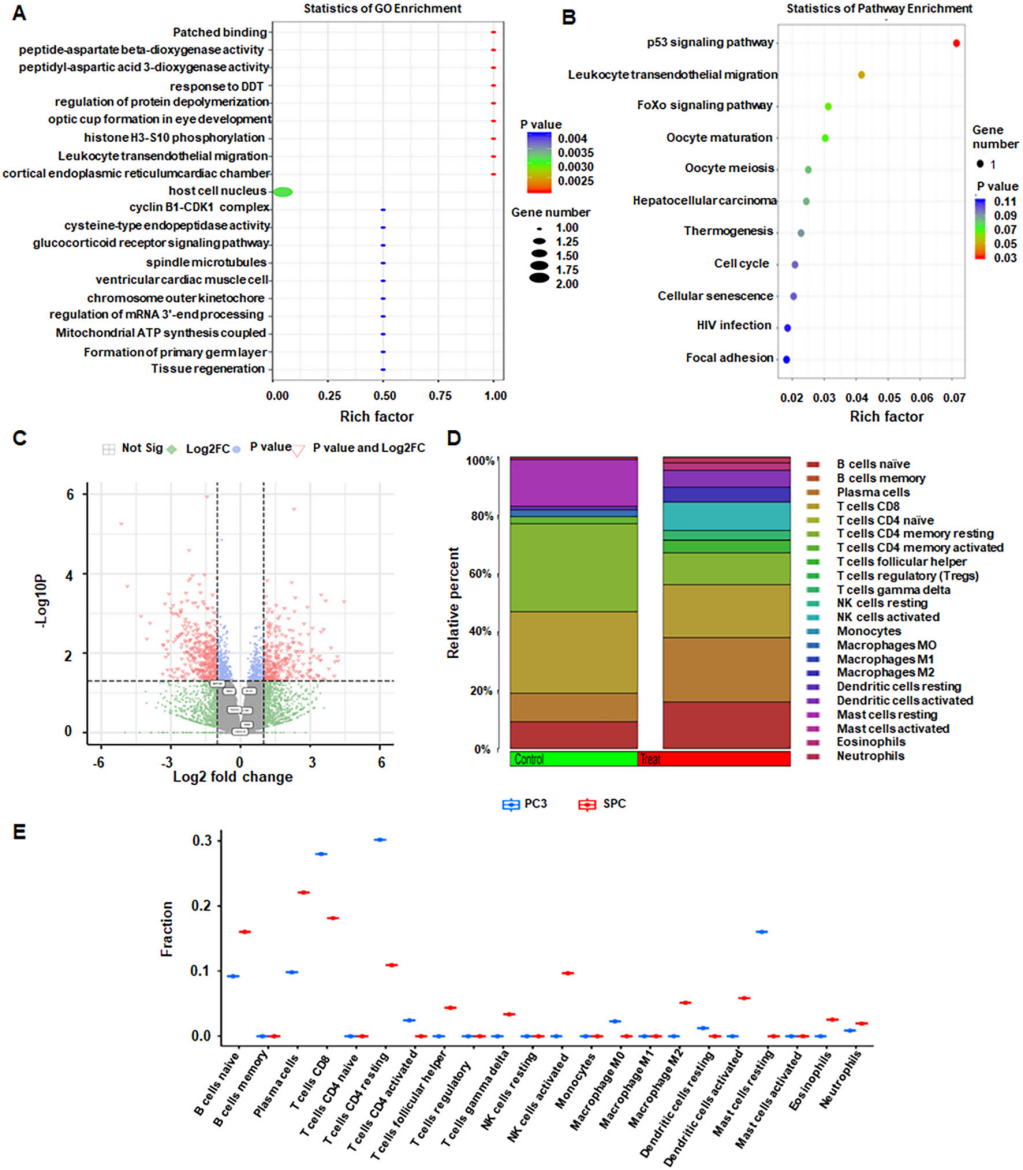
Transcriptomic analysis. **(A)** Gene Ontology (GO) enrichment of DEGs in SPC vs PC3. **(B)** Kyoto Encyclopedia of Genes and Genomes (KEGG) pathway enrichment bubble plot highlighting the most enriched pathways. **(C)** Volcano plot of DEGs. **(D)** Relative percentages of immune cell types in SPC and PC3. **(E)** Expression levels of 22 immune cell types between SPC and PC3 groups.

The biological processes, cellular components, and molecular functions associated with a list of genes of interest were investigated using GO enrichment analysis in SPC group (**Figure 1A**). Enrichment in biological processes includes regulation of transcription, DNA damage response, cell cycle arrest, cell division, blood vessel development and endocytosis (**Figure 1A**). Furthermore, enriched cellular components include the cytoplasm, nucleus, membrane, nucleoplasm, plasma membrane in SPC (**Figure 1A**). KEGG pathway analysis identified prominent enrichment of the p53 signaling and FoxO signaling pathways in SPC group (**Figure 1B**).

### 3.2 Differential expression between PC3 and SPC

Compared with controls, the SPC group exhibited 562 upregulated and 671 downregulated genes, with 5233 genes showing no significant change. A volcano plot summarizes the differential landscape (**Figure 1C**). Expression patterns of immune-related genes for control and SPC groups are displayed (**Figure 1D**). Immune-cell deconvolution revealed higher neutrophil infiltration in SPC, whereas CD4 T-cell abundance was greater in the control group (**Figure 1E**).

### 3.3 Biomarker selection with machine learning

**Figure 2:**
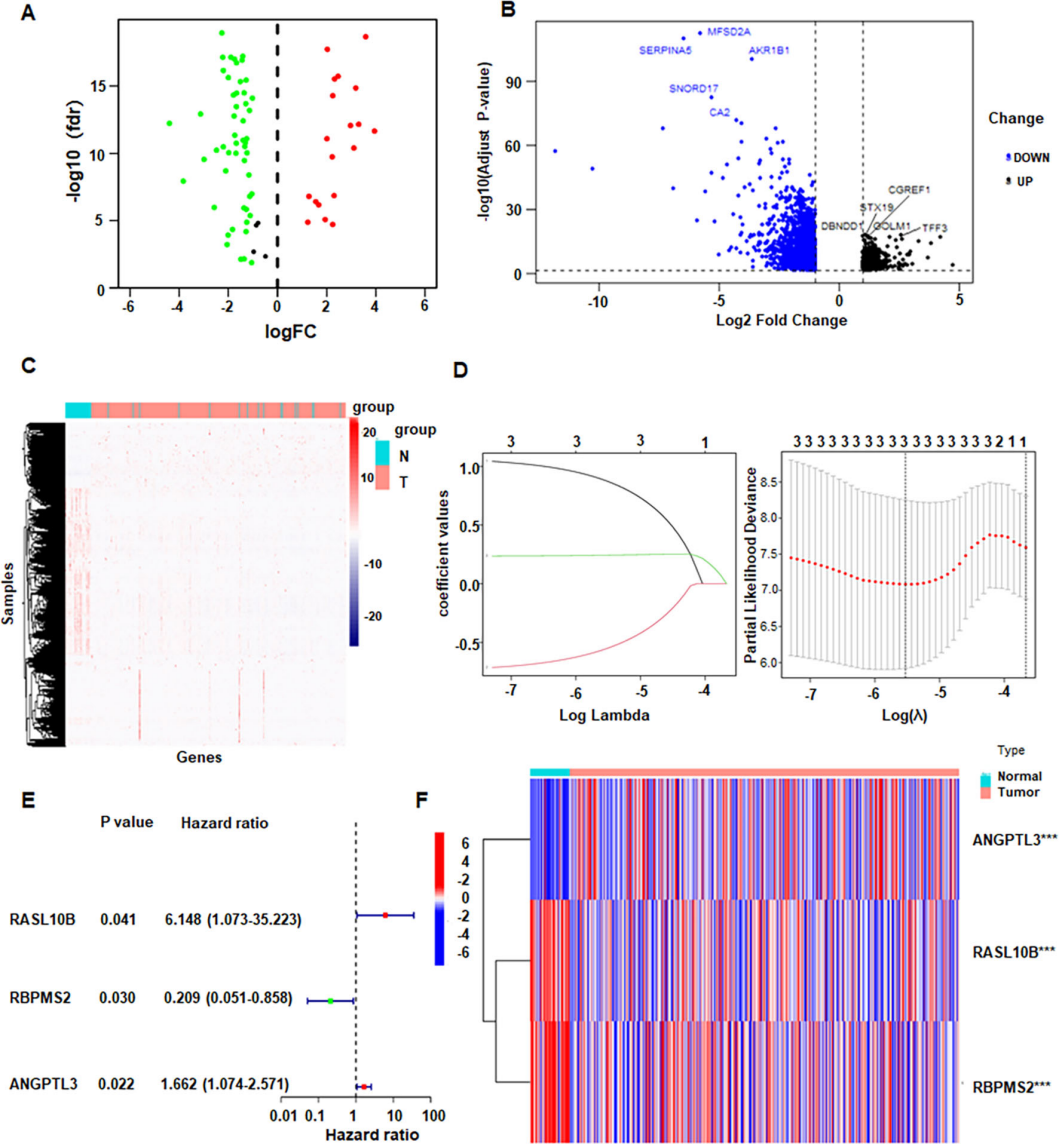
Expression profile in TCGA cohort. **(A)** Volcano plot of the 77 overlapping genes between tumor and normal samples. **(B)** Volcano plot with the top ten DEGs labeled. red: upregulated, blue: downregulated. **(C)** Heatmap of DEGS across samples. **(D)** Potential predictors from LASSO regression. **(E)** Forest plot of multivariate Cox regression analysis showing hazard ratios (HR) with 95% CIs for candidate genes. **(F)** Heatmap of the three-gene signature between tumor and normal groups.

**Figure 3:**
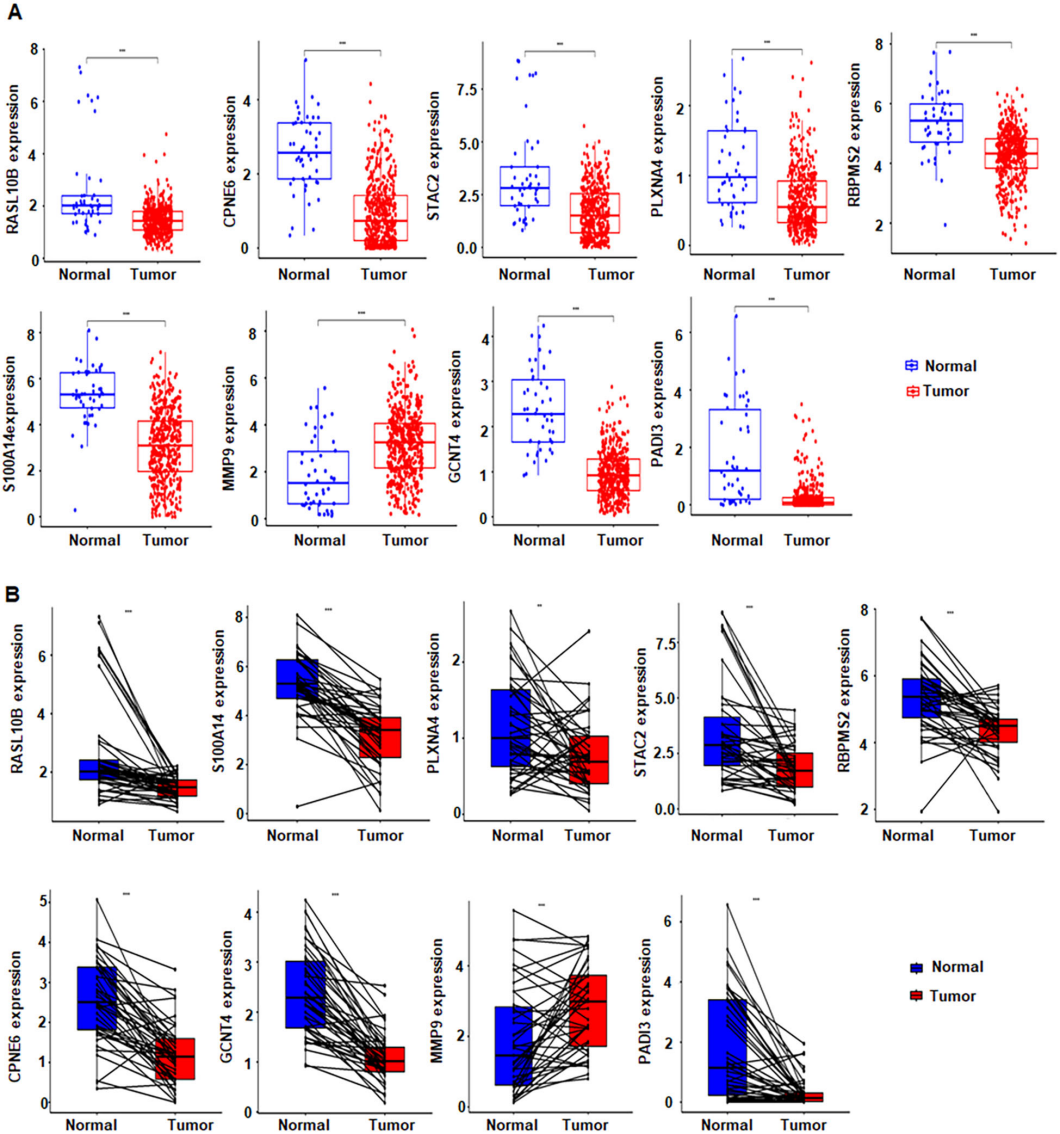
Gene expression in tissues. **(A)** Expression profiles of nine machine learning-selected genes in tumor and normal tissues. **(B)** Distribution comparing gene expression in paired tumor and adjacent normal tissues. **P < 0.01; ***P <0.001.

Several downregulated genes in SPC cells were identified (**Supplementary Figure S1A**). To integrate external evidence, we intersected our PC3/SPC RNA-seq results with TCGA data, yielding 77 overlapping genes (**Supplementary Figures S1B, C**). We then applied LASSO and SVM feature selection, and the consensus set comprised nine candidates, namely calcium-dependent phospholipid-binding protein 6 (CPNE6), RAS-like family member 10B (RASL10B), glucosaminyl (N-acetyl) transferase 4 (GCNT4), SH3 and cysteine-rich domain containing protein 2 (STAC2), RNA-binding protein with multiple splicing 2 (RBPMS2), peptidyl arginine deiminase 3 (PADI3), plexin A4 (PLXNA4), S100 calcium-binding protein A14 (S100A14), and matrix metallopeptidase 9 (MMP9) (**Supplementary Figure S1D**). Expression analysis demonstrated MMP9 upregulated in tumors, whereas the other eight genes were reduced. Differential analysis across the 77 overlapping genes is summarize (**Figure 2A**). A volcano plot revealed the top 10 DEGs, which were major facilitator superfamily domain containing 2A (MFSD2A), serpin family A member 5 (SERPINA5), Aldo-keto reductase family 1 member B1 (AKR1B1), small nucleolar RNA, C/D box 17 (SNORD17), cell growth regulator with EF-hand domain 1 (CGREF1), syntaxin 19 (STX19), DENN domain containing 1 (DENDD1), Golgi membrane protein 1 (GOLM1), trefoil factor 3 (TFF3), and carbonic anhydrase 2 (CA2) (**Figure 2B**). A heatmap showed their distribution in normal versus tumor groups (**Figure 2C**). LASSO shrank the feature set from 77 to three predictors, with coefficient paths and cross-validated error (**Figure 2D**). Based on Cox regression, we constructed a three-gene prognostic model that included RASL10B, RBPMS2, and angiopoietin-like 3 (ANGPTL3) (**Figure 2E**). A gene expression heatmap was generated to visualize the expression levels of three genes (**Figure 2F**), whose components were significantly associated with patient prognosis (**Figure 3A**). Box plots compare expression patterns between normal and tumor samples (**Figure 3B**).

### 3.4 Identification of ubiquitination-related biomarker genes

**Figure 4:**
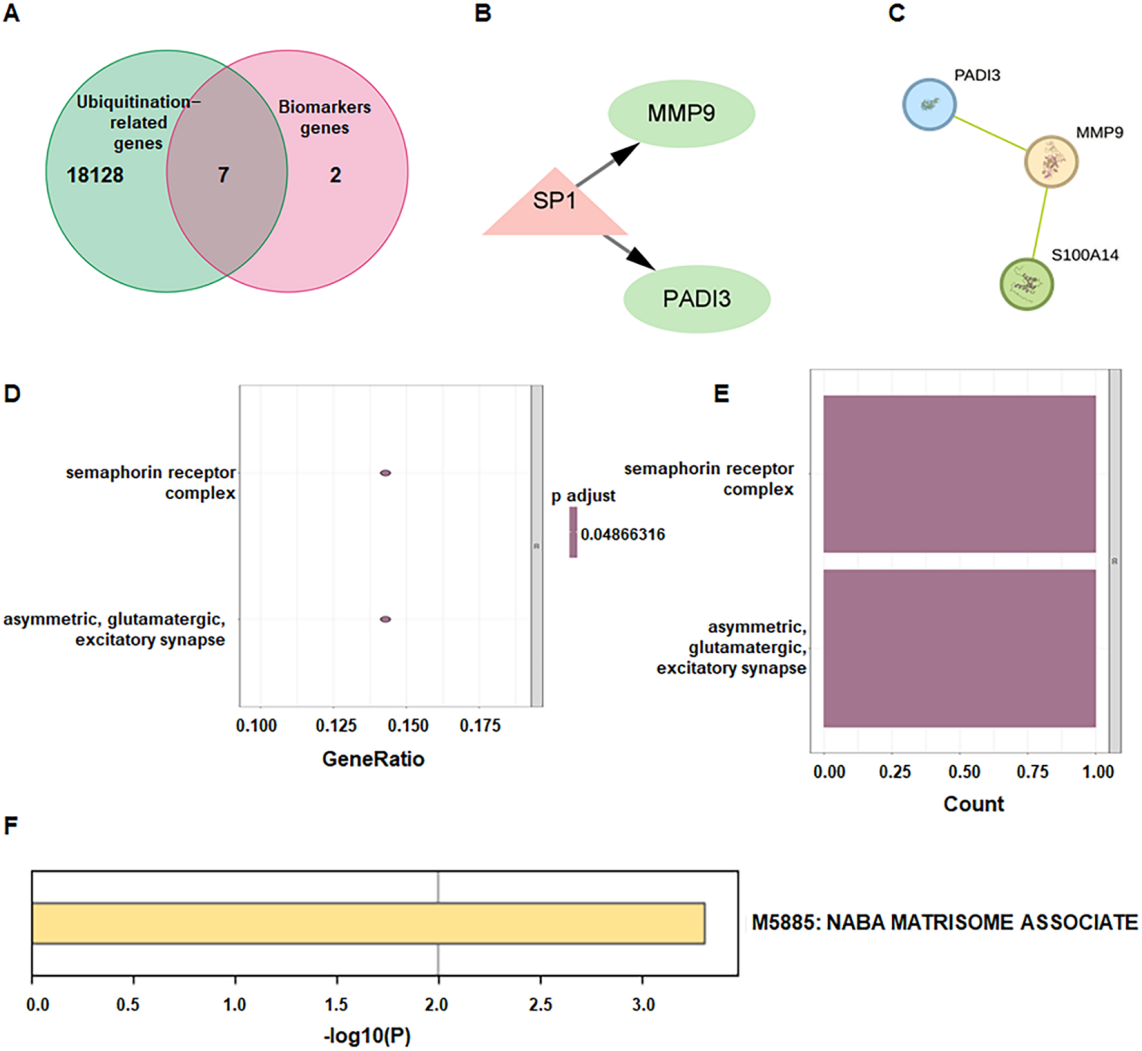
Identification and characterization of ubiquitination−related biomarker genes. **(A)** Venn diagram showing the overlap between ubiquitination−related genes and biomarker genes (UR-BGs). **(B)** Transcription factor (TF) analysis for the 7 UR-BGs. **(C)** Protein-protein interaction networks (PPI) of the 7 UR-BGs. **(D)** Bar graph of GO enrichment for the 7 UR-BGs. **(E)** Bubble plot of GO cellular component enrichment. **(F)** Bar graph of enriched terms across UR-BGs, colored by p-values.

From GeneCards database, 18, 135 URGs were retrieved, and intersection analysis yields seven ubiquitination-related biomarkers (UR-BGs), including MMP9, RASL10B, RBPMS2, S100A14, PLXNA4, CPNE6, and PADI3 (**Figure 4A**). In the transcription-factor network, SP1 was predicted to regulate MMP9 and PADI3 (**Figure 4B**). For STRING PPI analysis (minimum confidence 0.15; disconnected nodes hidden), a three-gene module comprising PADI3, MMP9, S100A14 was retained (**Figure 4C**). GO enrichment of the seven UR-BGs highlighted cellular components including the semaphorin receptor complex and asymmetric/glutamatergic excitatory synapse (**Figures 4D, E**). Metascape further indicated enrichment in M5885: NABA MATRISOME ASSOCIATED, implicating extracellular-matrix remodeling (**Figure 4F**).

### 3.5 Multi-layer gene and drug-gene interaction network

**Figure 5:**
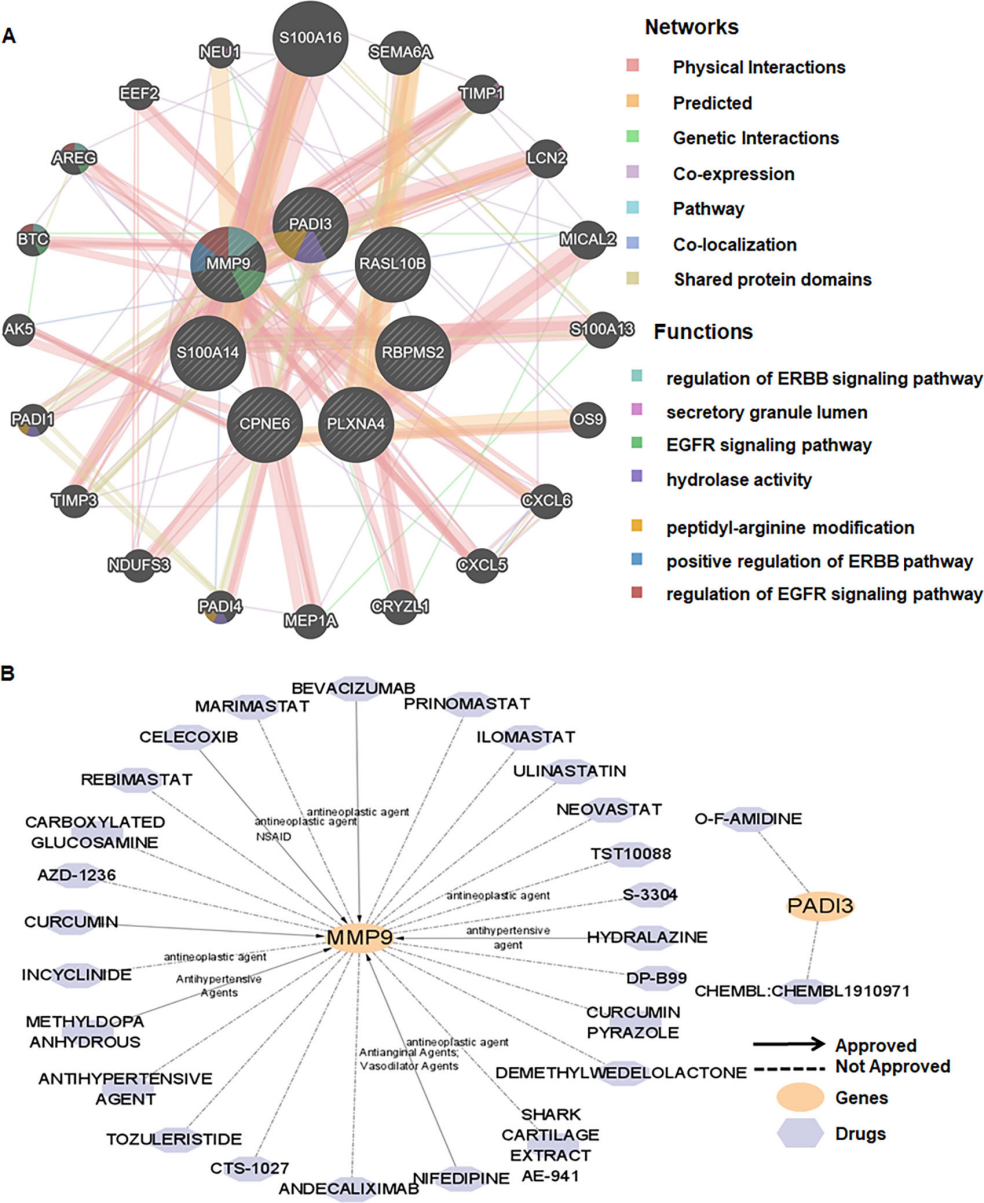
Gene function and drug-gene interaction networks for seven UR-BGs. **(A)** Gene function prediction based on GeneMANIA database. **(B)** DGIdb drug-gene interaction network highlighting investigational agents.

GeneMANIA linked the seven UR-BGs to 20 related genes, mapping functions such as ERBB/EGFR signaling regulation, secretory granule lumen, peptidyl-arginine modification, and positive regulation of ERBB signaling (**Figure 5A**). DGIdb predicted drug–gene interactions for MMP9 and PADI3. In addition, 25 agents targeted MMP9, including six approved drugs spanning antihypertensive and antineoplastic classes, while O-F-AMIDINE and CHEMBL1910971 were predicted for PADI3 but are not approved (**Figure 5B**). These networks nominate actionable nodes within ubiquitination-linked pathways for therapeutic exploration.

### 3.6 Predicted for ubiquitin ligase-substrate interactions

An E3–substrate prediction network was constructed for the seven UR-BGs (**Supplementary Figure S2**). At the database’s default confidence, S100A14 and PADI3 lacked confident E3 assignments. For MMP9, predicted E3 candidates were membrane associated Ring-CH-type finger 6 (MARCHF6), MARCHF11, MARCHF1, MARCHF8, and MARCHF3. For PLXNA4, candidates included Cbl proto-oncogene (CBL), CBLC, CBLB, neuralized E3 ubiquitin protein ligase 1 (NEURL1), and Zinc finger MYND-type containing 8 (ZMYND8). Network hubs connecting to multiple UR-BGs comprised parkin RBR E3 ubiquitin protein ligase (PRKN), neural precursor cell expressed, developmentally down-regulated 4-like (NEDD4L), CBL, ariadne RBR E3 ubiquitin protein ligase 1 (ARIH1), midline 1 (MID1), MID2, promyelocytic leukemia (PML), and synaptotagmin-like 4 (SYTL4). CPNE6 and RASL10B shared predicted E3/adaptor candidates (SYTL4, MARCHF6, FBXO5, MARCHF5), whereas RBPMS2 linked to BTRC. These predictions nominate testable E3–substrate axes for experimental validation.

### 3.7 Construction of a prognostic model in PCa

**Figure 6:**
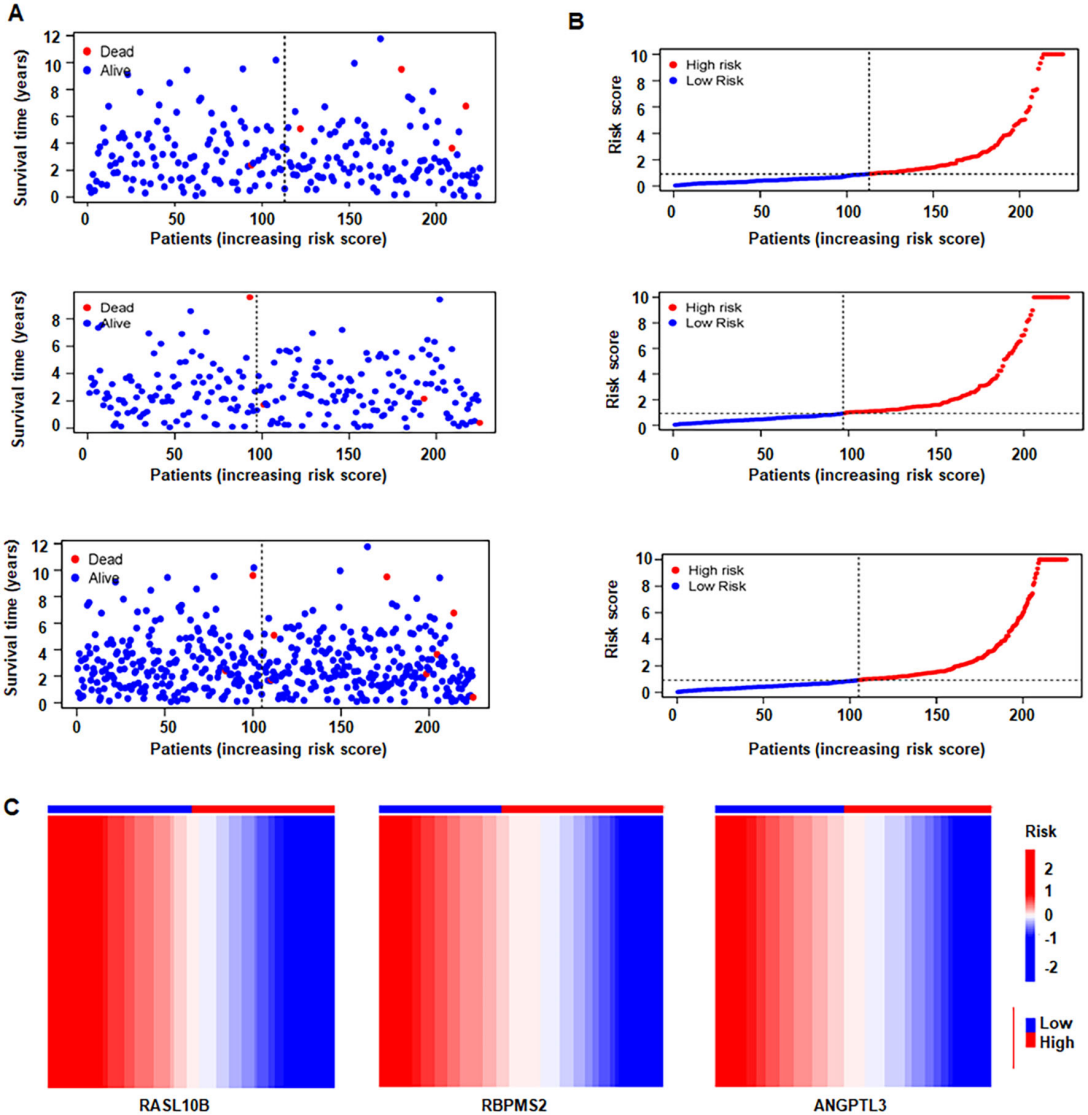
Survival and risk stratification in prostate cancer. **(A)** Survival status plot ordered by risk score. Red means deceased patients, while blue means alive patients. **(B)** A distributions of risk scores. Red means high-risk score, while blue means low-risk score. **(C)** Heatmap of the three-gene signature across risk groups. Red represents high expression, while blue represents low expression.

Clinico-pathologic association analyses showed that RASL10B correlated with T and N stage (**Supplementary Figure S3**). CPNE6 was associated with T/N stage, suggesting relevance to primary tumor burden and nodal status. Patients were then stratified into high- and low-risk groups based on the median risk score derived from the overlapping-gene set. Kaplan–Meier curves demonstrated divergent survival between strata (**Figure 6A**), supported by cohort-wide survival analysis, a risk-ordered heatmap, and prognostic evaluation (**Figures 6B, C**). Together, these results indicate that the selected genes, particularly RASL10B and CPNE6, carry prognostic information and that the resulting risk signature can stratify outcomes in PCa.

### 3.8 Immune microenvironment analysis

**Figure 7:**
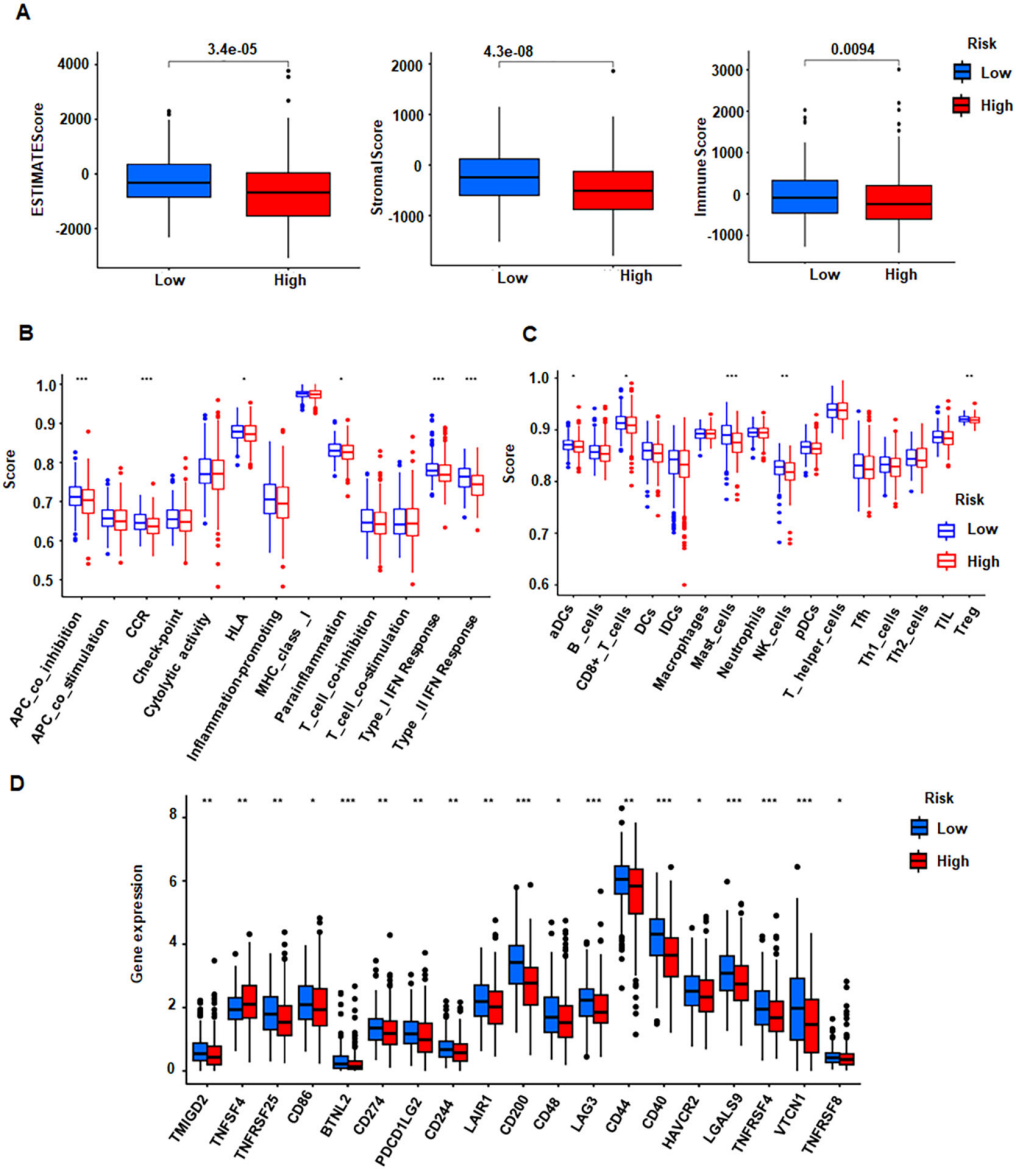
Analysis of immune microenvironment by risk group. **(A)** ESTIMATE, stromal, and immune scores compared between high- and low-risk groups. **(B)** Boxplots of immune function scores in low vs high risk. **(C)** Boxplots of immune checkpoints scores in low vs high risk. **(D)** Boxplots of checkpoint gene expressions in low vs high risk. *P <0.05; **P < 0.01; ***P <0.001.

Using ESTIMATE, we inferred stromal, immune, and composite ESTIMATE scores from bulk expression data. The low-risk cohort displayed significantly higher immune, stromal, and ESTIMATE scores than the high-risk group (**Figure 7A**), indicating a more inflamed, stroma-rich tumor microenvironment that is often associated with improved outcomes in PCa. Consistently, C-C chemokine receptor (CCR) expression differed between risk groups (**Figure 7B**), with lower CCR levels in the high-risk cohort (**Figure 7C**). Multiple immune checkpoint genes, such as CD274 (PD-L1), also showed differential expression between groups (**Figure 7D**), further underscoring immune-context differences that may bear therapeutic relevance.

### 3.9 Drug sensitivity analysis

In silico drug-response profiling revealed differential sensitivity between risk groups. The high-risk cohort exhibited greater predicted sensitivity to a panel of agents, including AUY922, AP.24534, AICAR, AG014699, A.770041, OSI.906, PF.02341066, docetaxel, embelin, and dasatinib (**Supplementary Figures S4, S5**). These findings suggest that molecular features captured by the risk signature may inform therapeutic selection and identify candidates for risk-adapted treatment strategies.

### 3.10 Analysis of single cell sequencing

**Figure 8:**
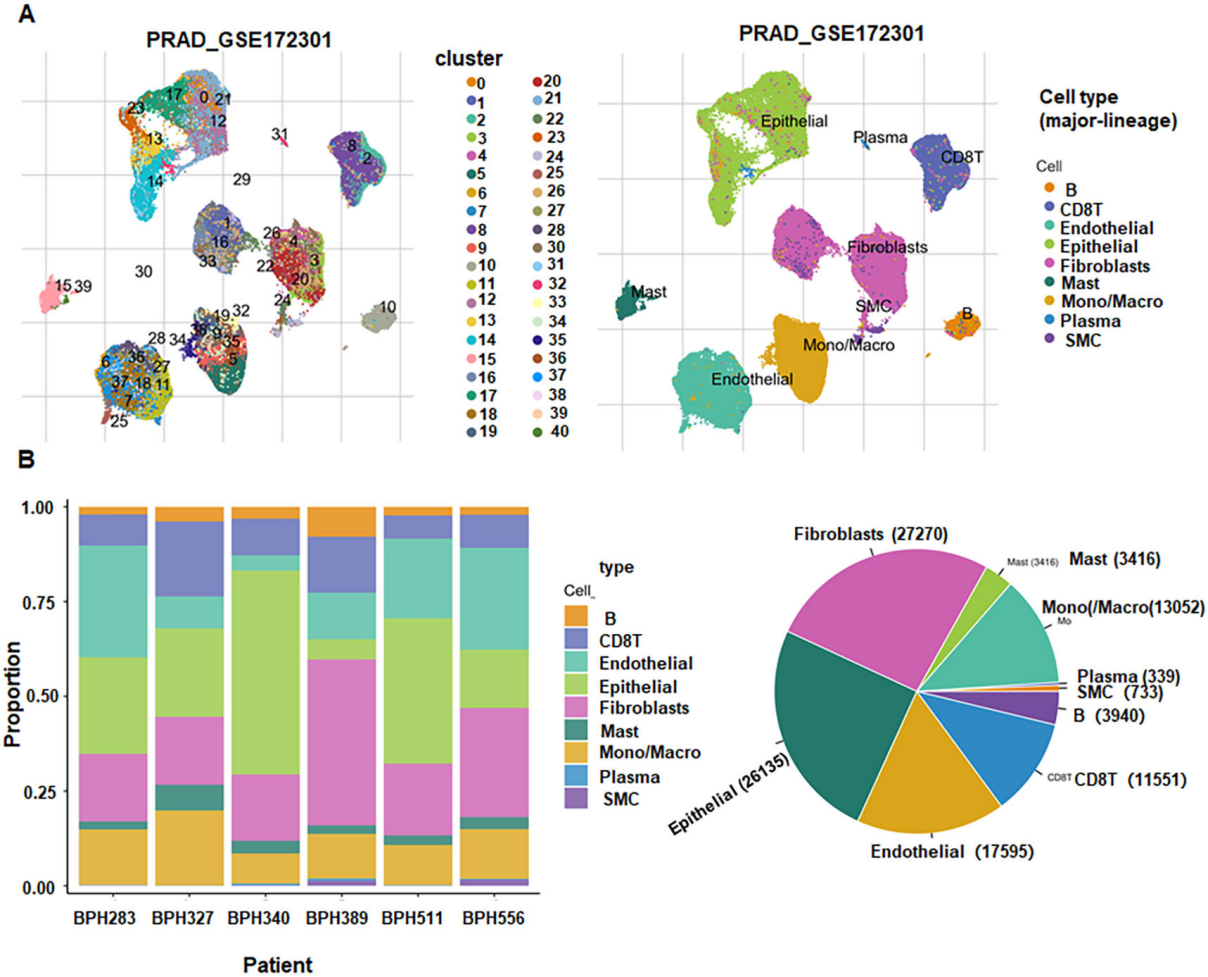
Single-cell sequencing analysis. **(A)** Clustering of GSE172301 into 40 clusters with cell-type annotations. **(B)** Per-patient cell-type composition (pie charts), showing proportional abundance of annotated cell types.

**Figure 9:**
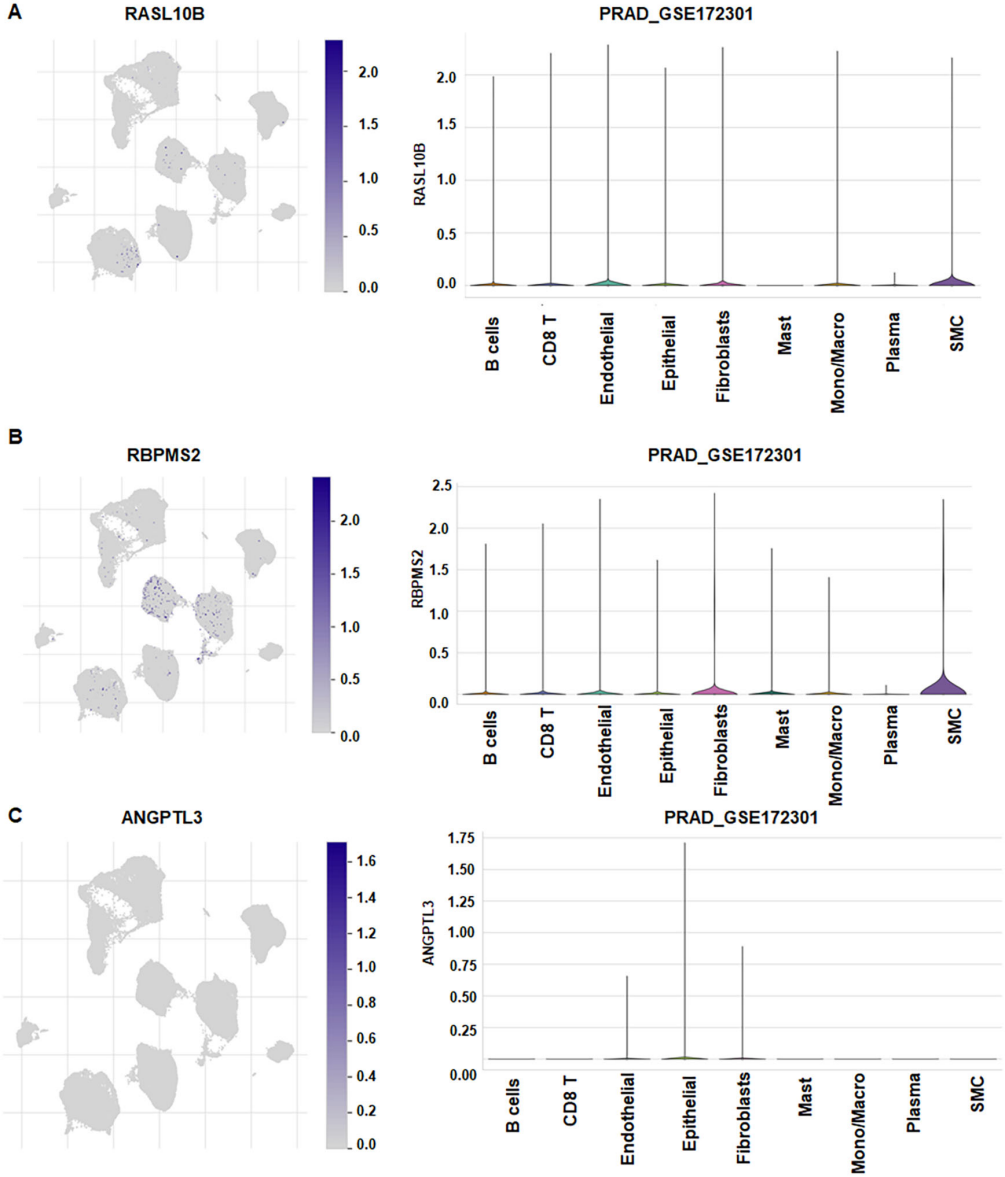
Cell-type expression of RASL10B, RBPMS2 and ANGPTL3. **(A)** Expression of RASL10B across various cell types. **(B)** Expression of RBPMS2 across various cell types. **(C)** Expression of ANGPTL3 across various cell types.

Using GSE172301, we performed single-cell annotation and identified 40 clusters (**Figure 8A**), which were consolidated into nine cell types: B cells, CD8 T cells, endothelial cells, epithelial cells, fibroblasts, mast cells, monocytes/macrophages, plasma cells, and smooth muscle cells (SMC) (**Figure 8B**). Cell-type composition varied across samples; for example, BPH389 was enriched for fibroblasts, whereas BPH340 showed a higher epithelial fraction. Gene-level mapping revealed broad RASL10B expression across epithelial cells, SMCs, endothelial cells, fibroblasts, CD8 T cells, and B cells (**Figure 9A**). RBPMS2 was detectable in B cells, CD8 T cells, endothelial, epithelial, fibroblast, mast, monocyte/macrophage, and SMC compartments, with the highest signal in SMCs (**Figure 9B**). ANGPTL3 expression was largely restricted to endothelial, epithelial, and fibroblast populations (**Figure 9C**).

### 3.11 Downregulation of PLXNA4 inhibits cell viability and proliferation

**Figure 10:**
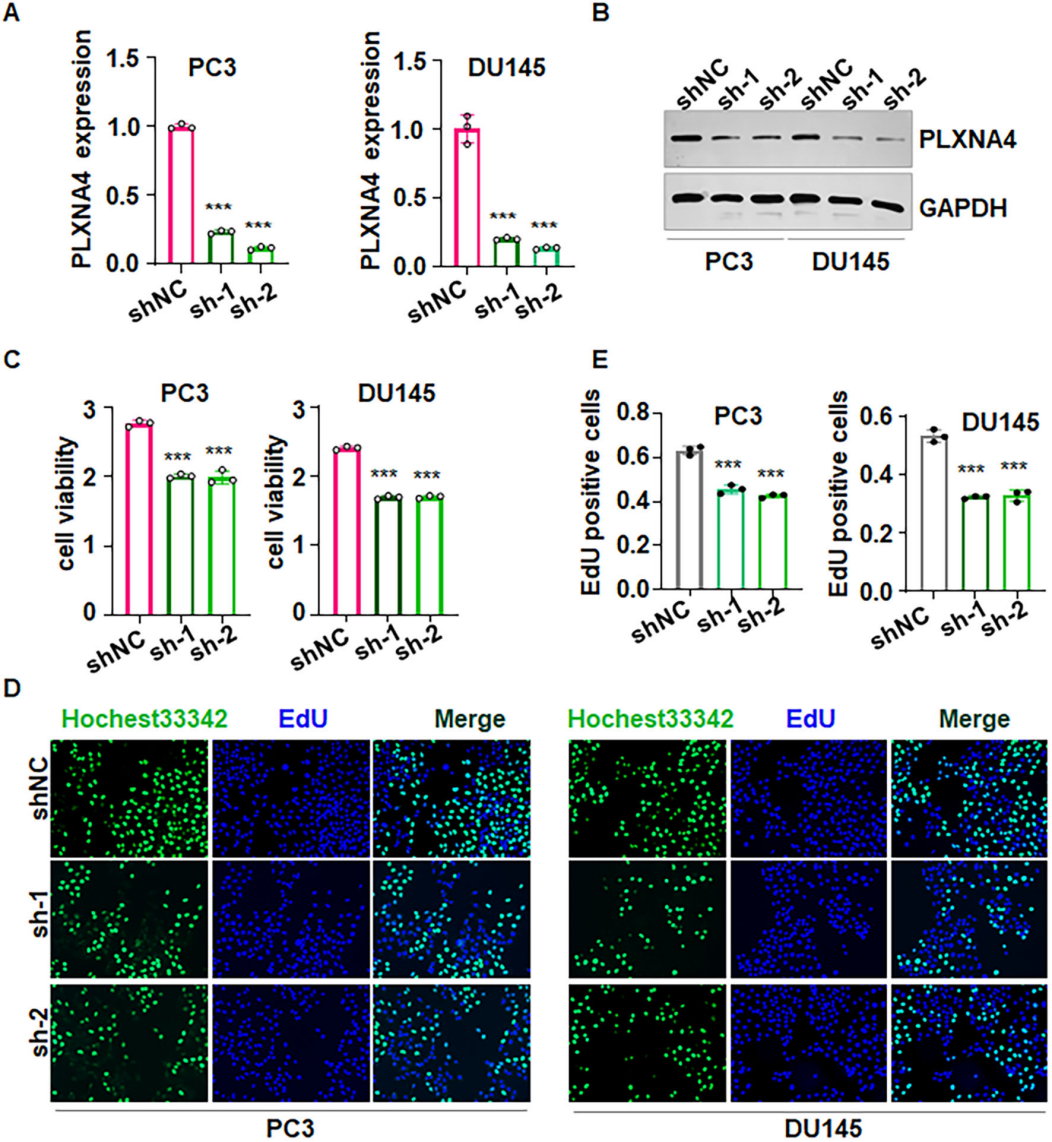
PLXNA4 downregulation reduces viability and proliferation. **(A)** RT-PCR showing decreased PLXNA4 mRNA in PC3 and DU145 cells transfected with sh-PLXNA4. shNC: shRNA control; sh-1: sh-PLXNA4-1; sh-2: sh-PLXNA4-2. **(B)** Western blot confirming reduced PLXNA4 protein after shRNA transfection. **(C)** CCK-8 assays indicating diminished cell viability upon PLXNA4 silencing. **(D)** Representative EdU images demonstrating fewer EdU-positive nuclei after PLXNA4 knockdown. Nuclei were counterstained with Hoechst 33342. **(E)** Quantification of EdU incorporation showing reduced proliferating cells following PLXNA4 depletion. Data are mean ± SD. ***P < 0.001 versus shNC.

Our RT-PCR analysis confirmed efficient PLXNA4 knockdown in PC3 and DU145 cells transfected with shRNAs compared with shNC (**Figure 10A**). Furthermore, Western blotting validated reduced PLXNA4 protein in PCa cells upon shRNA transfection (**Figure 10B**). Moreover, CCK-8 assays demonstrated a significant decline in cell viability after PLXNA4 silencing in both cell lines (**Figure 10C**). Notably, EdU immunofluorescence images demonstrated fewer EdU-positive nuclei upon PLXNA4 depletion (**Figure 10D**). Quantification of EdU incorporation confirmed a significant decrease in the percentage of proliferating cells upon PLXNA4 depletion (**Figure 10E**). Therefore, loss of PLXNA4 compromises PCa cell viability and proliferation.

### 3.12 Downregulation of PLXNA4 induces apoptosis

**Figure 11:**
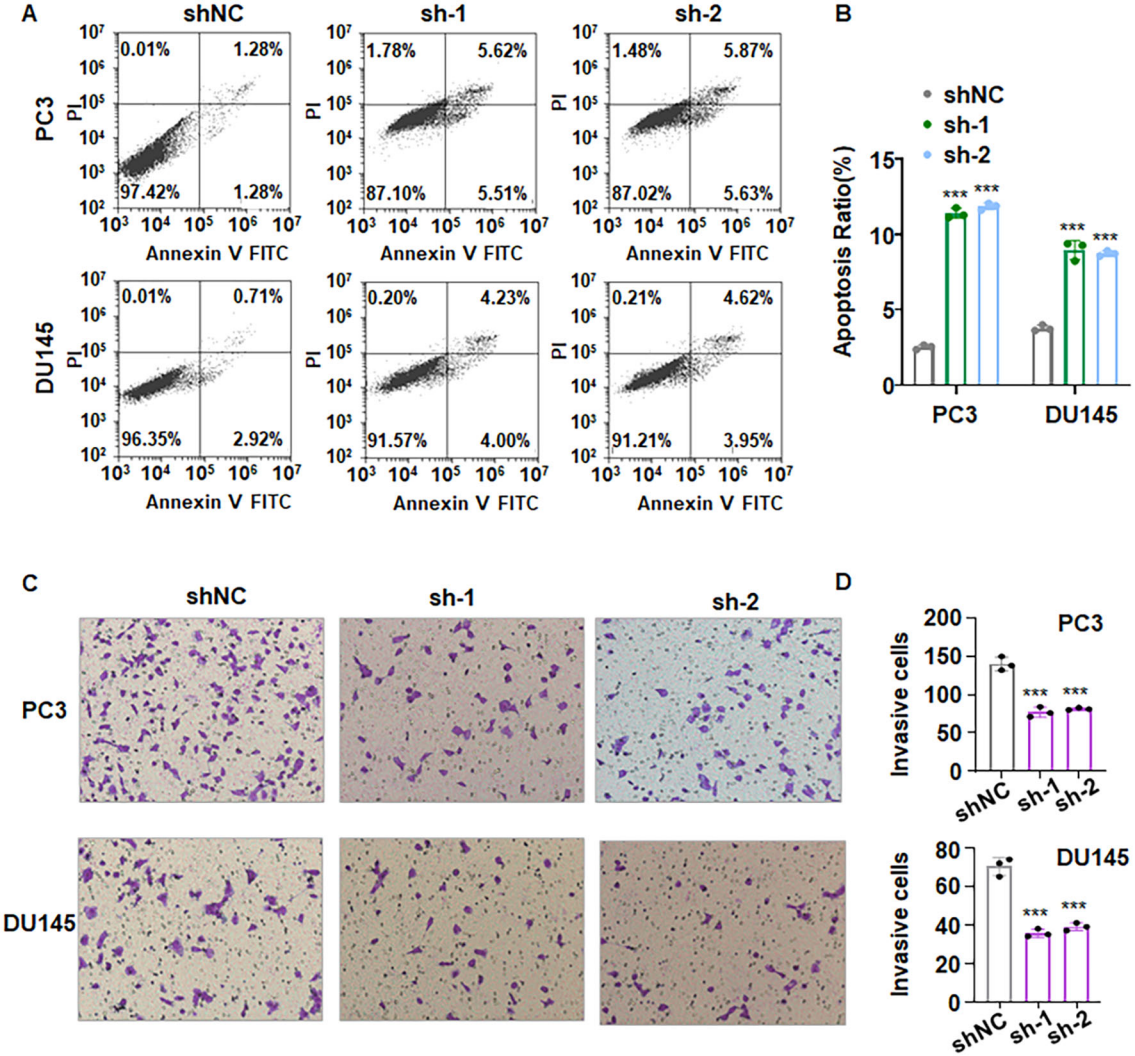
PLXNA4 downregulation induces apoptosis and suppresses invasion. **(A)** Representative Annexin V-FITC/PI flow-cytometry plots of PC3 and DU145 cells transfected with sh-PLXNA4 or shNC. shNC: shRNA control; sh-1: sh-PLXNA4-1; sh-2: sh-PLXNA4-2. **(B)** Quantification of total apoptotic fractions (early + late) shows significant increases with both shRNAs. Data are mean ± SD. ***P < 0.001 versus shNC. **(C)** Representative micrographs from Matrigel-coated Transwell assays showing markedly decreased invasive ability after PLXNA4 silencing in PC3 and DU145 cells. **(D)** Quantification confirms a robust reduction in invaded cells. Data are presented as mean ± SD; ***P < 0.001 versus shNC.

To determine whether downregulation of PLXNA4 inhibits cell proliferation due to influencing apoptotic death, we performed Annexin V-FITC/PI flow cytometry to measure apoptotic cells in PCa. Our results revealed a marked rise in total apoptotic fraction (early + late) in PC3 and DU145 cells after PLXNA4 depletion (**Figure 11A**). In PC3 cells, apoptosis increased from ~2.5% in shNC to ~11.1% (sh-1) and ~11.5% (sh-2), respectively. In DU145 cells, apoptosis rose from ~3.6% in shNC to ~8.2% (sh-1) and ~8.6% (sh-2), respectively (**Figure 11B**). Hence, PLXNA4 depletion robustly triggered apoptosis in PCa cells.

### 3.13 Downregulation of PLXNA4 inhibits invasion

To assess invasive behavior, Matrigel-coated Transwell assays were conducted following PLXNA4 silencing in PC3 and DU145 cells. Both shRNAs produced a pronounced reduction in invaded cells relative to shNC, with decreases of roughly 40–50% in both PC3 and DU145 cells (**Figures 11C, D**). Collectively, these data indicate that loss of PLXNA4 markedly compromises the invasive capacity of human PCa cells, supporting a pro-invasive role for PLXNA4.

## 4 Discussion

Several studies have utilized bulk and scRNA-seq to explore mechanisms of PCa tumorigenesis. One study maps epithelial-mesenchymal transition (EMT)-related DEGs in PCa, validates findings in TCGA, and highlights ECM-linked biomarkers for diagnosis and therapeutic targeting, including integrin subunit beta like 1 (ITGBL1), desmocollin 3 (DSC3), collagen type IV alpha 6 chain (COL4A6), angiopoietin 1 (ANGPT1), armadillo repeat containing, X-linked 1 (ARMCX1), microtubule associated monooxygenase, calponin and LIM domain containing 2 (MICAL2), EPH receptor A5 (EPHA5) (43). Another study reported that phosphatase and tensin homolog (PTEN) loss in basal prostate cells triggers regional, immune-linked plasticity, reprogramming to hillock-like then proximal-like luminal states that seed invasive tumors (44). Collagen I upregulation and increased ECM stiffness promote adult basal stem-cell multipotency in mammary and prostate epithelia, mediated by a β1-integrin/focal adhesion kinase (FAK)/activator protein 1 (AP-1) axis (45). Matrix choice dictates prostate patient-derived organoids fidelity. Matrigel-free cultures retain AR-active tumor heterogeneity and intermediate cells, whereas Matrigel drives basal-like overgrowth (46). By integrating our sequencing data and TCGA, we identified 77 overlapping genes for differential analysis and functional annotation. KEGG and GO enrichment highlighted pathways central to tumor biology, including p53 signaling pathway. It is known that p53 pathway is essential for preserving genome integrity and stopping the growth of tumors (47–49). Our study revealed that MMP9 is significantly more expressed in tumor cells. It is well documented that MMP9 is a critical factor in promoting tumor metastasis in PCa (50). In line with our study, S100A14 was found to suppress cell growth and EMT via targeting Hippo signaling pathway in PCa (51).

Our analysis revealed that CPNE6 is significantly correlated with the clinical staging of T and N, indicating its potential role in predicting tumor size and lymph node involvement. Additionally, CPNE6 was found to be differentially expressed in tumor cells, suggesting its potential role in cancer development and progression (52). CPNE6 belongs to the copine family of calcium-dependent phospholipid-binding proteins, which have been implicated in a variety of cellular processes, including signal transduction, cell differentiation, and apoptosis (53). In addition, CPNE6 has been found to be overexpressed in several types of cancers, including GBM (54), endometrial cancer (55), and lung cancers (53). However, the role of CPNE6 in PCa has not been well-studied.

RBPMS2 is an RNA-binding protein that has been shown to regulate gene expression at the post-transcriptional level (56). RBPMS2 is upregulated in various types of cancer (57, 58). In gastric cancer, RBPMS2 is overexpressed, predicts poor prognosis, and promotes proliferation, invasion, and migration by suppressing NLRP3/caspase-1/GSDMD-mediated pyroptosis (59). However, the exact role of RBPMS2 in PCa remains unclear. In this study, RBPMS2 was associated with prognosis in PCa. Similarly, previous study demonstrated significant association of RBPMS2 with PCa risk (60). STAC2 has been previously identified as a potential tumor suppressor gene in various cancers, including colorectal, and breast cancer (61, 62). Our results demonstrated that the expression of STAC2 was significantly decreased in PCa tissues compared to adjacent normal tissues, which is consistent with previous studies on other types of cancers (63, 64). These results indicate that STAC2 may also play a role in the development and progression of PCa. Additionally, we observed an association between low STAC2 expression and higher tumor grade and advanced clinical stage, suggesting that STAC2 may serve as a prognostic marker for PCa. CHD4 induces PADI1 and PADI3, causing pyruvate kinase isozyme M2 (PKM2) R106 citrullination that enhances serine activation, sustaining glycolysis under hypoxia and reshaping cancer cell proliferation (65). GCNT4 is markedly downregulated in gastric cancer, with low expression associating with poorer OS and DFS. GCNT4 overexpression suppresses cell proliferation by arresting the cell cycle in gastric cancer (66). RASL10B is a widely expressed, cytoplasmic Ras-like small GTPase whose mRNA is downregulated in breast cancer cells (67). RASL10B is methylated in sessile serrated adenoma/polyp (SSA/P) and cancer in SSA/P (67). From TCGA, 167 DEGs distinguish left/right colon cancers. Prognostic models identified phosphatase and actin regulator 3 (PHACTR3)/creatine kinase, mitochondrial 2 (CKMT2) for left and epiregulin (EREG)/erythroferrone (ERFE)/growth factor independent 1 (GFI1)/RASL10B for right, with right-sided DEGs enriched for immune-related pathways (68). Across cancers, higher succinylation scores aligned with oxidative phosphorylation and lower scores with immune differentiation. An 11-gene signature, including RASL10B, predicted poorer survival in colon cancer (69). ANGPTL3 increases sorafenib sensitivity by inhibition of SNAI1 and CPT1A in liver cancer (70). ANGPTL3 levels have not remarkedly change in locally advanced PCa patients (71). Without a doubt, it is required to explore the functions of PADI3, GCNT4, RASL10B, and ANGPTL3 in PCa progression.

PLXNA4 is a transmembrane receptor in the class-A plexin family that transduces semaphorin cues. It binds sema6 and sema3 via neuropilin-1/2 co-receptors. In non-small cell lung cancer (NSCLC), miR-564 is directly targets the PLXNA4, leading to suppression of proliferation, migration, invasion, and tumor growth (72). PLXNA4 forms complexes with fibroblast growth factor receptor 1 (FGFR1) and vascular endothelial growth factor receptor 2 (VEGFR-2) to boost basic fibroblast growth factor (bFGF)/VEGF pathways, promoting proliferation and tumor growth (73). A recent study has demonstrated that targeting PLXNA4 may offer a promising avenue for enhancing the efficacy of immune checkpoint blockade therapy in cancer treatment of melanoma (74). More specifically, PLXNA4 plays a role in the tumor microenvironment by negatively regulating the migration and proliferation of cytotoxic T cells (CTLs), thus limiting their potential to infiltrate tumors and limit cancer progression (74). B7-H4Ig suppresses inflammatory CD4^+^ T-cell responses by binding Sema3A, which bridges to an NRP1/PLXNA4 complex that elevates phosphorylated PTEN, thereby enhancing Foxp3^+^ T-reg cell numbers and function (75). Our findings showed that PLXNA4 promotes cell proliferation and invasion in PCa cells, suggesting that PLXNA4 expression can serve as a potential target for Pca.

In mCRPC models, immune checkpoint blockade alone or myeloid-derived suppressor cells (MDSC)-targeted therapy is weak, but their combination synergistically suppresses tumors by reprogramming cytokines and neutralizing MDSC-mediated immunosuppression (76). Single-cell profiling identifies SPP1^hi^ tumor-associated macrophages as drivers of ICI resistance in mCRPC. Adenosine A2A receptor blockade depletes SPP1^hi^-TAMs, restores CD8^+^ function, and sensitizes tumors to PD-1/PD-L1 inhibitors (77). In our study, several immune checkpoint genes, including CD274 (PD-L1), were differentially expressed between the risk groups, highlighting distinct immune contexts that could have important therapeutic implications.

## 5 Conclusion

This study integrates bulk, single-cell/spatial transcriptomics and clinical data to nominate biomarkers and therapeutic targets in PCa. We identified nine candidates and derived a risk signature that stratifies patient outcomes, alongside immune-context differences between risk groups. Functional assays established PLXNA4 as a pro-proliferative, pro-invasive factor, underscoring its potential as a therapeutic vulnerability. Collectively, these findings support the diagnostic and prognostic utility of the proposed markers and highlight immune and ubiquitin-pathway biology as actionable axes for personalized therapy. Several limitations should be mentioned. For example, it is better to consider validating SPC signatures in patient-derived organoids or laser-capture micro-dissected PCa epithelium. Both PC3 and DU145 are androgen receptor–negative cell lines. It would be preferable to include AR-positive cells, such as C4-2, LNCaP, to test whether androgen signaling regulates PLXNA4 expression. This study assessed PLXNA4 function only *in vitro* and did not perform *in-vivo* validation or analyze clinical specimens. Future work incorporating *in vivo* experimental and clinical samples will be necessary to substantiate role of PLXNA4 in prostate tumorigenesis. Moreover, there is currently no robust evidence that PLXNA4 expression predicts survival in PCa. Cohort-specific validation by immunohistochemistry on well-annotated tumor microarray with survival endpoints will be necessary to establish prognostic value and to support PLXNA4 as a PCa biomarker.

## Data Availability

The data presented in the study are deposited in the https://ngdc.cncb.ac.cn repository, accession number PRJCA053727.

## Glossary

ABCG2: ATP binding cassette subfamily G member 2
AKR1B1: Aldo-keto reductase family 1 member B1
ANGPT1: angiopoietin 1
ANGPTL3: angiopoietin-like 3, AP-1, activator protein 1
AR: androgen receptor
ARIH1: ariadne RBR E3 ubiquitin protein ligase 1
ARMCX1: armadillo repeat containing, X-linked 1
bFGF: basic fibroblast growth factor
CA2: carbonic anhydrase 2
CBL: Cbl proto-oncogene
CCR: C-C chemokine receptor
CGREF1: cell growth regulator with EF-hand domain 1
CHD4: chromodomain helicase DNA binding protein 4
CKMT2: creatine kinase, mitochondrial 2
COL4A6: collagen type IV alpha 6 chain
CPNE6: calcium-dependent phospholipid-binding protein 6
CSC: cancer stem cell
CTLs: cytotoxic T cells
DEG: differentially expressed gene
DENDD1: DENN domain containing 1
DGIdb: drug-gene interaction database
DSC3: desmocollin 3
EMT: epithelial-mesenchymal transition
EPHA5: EPH receptor A5
EREG: epiregulin
ERFE: erythroferrone
FAK: focal adhesion kinase
FGFR1: fibroblast growth factor receptor 1
GCNT4: glucosaminyl (N-acetyl) transferase 4
GFI1: growth factor independent 1
GO: gene ontology
GOLM1: Golgi membrane protein 1
ITGBL1: integrin subunit beta like 1
KEGG: kyoto encyclopedia of genes and genomes
LASSO: least absolute shrinkage and selection operator
MARCHF6: membrane associated Ring-CH-type finger 6
MDSC: myeloid-derived suppressor cells
MFSD2A: major facilitator superfamily domain containing 2A, MHC-1, major histocompatibility complex class 1
MID1: midline 1
MMP9: matrix metallopeptidase 9
NEDD4L: neural precursor cell expressed, developmentally down-regulated 4-like
NEURL1: neuralized E3 ubiquitin protein ligase 1
NGS: next-generation sequencing
PADI3: peptidyl arginine deiminase 3
PCa: prostate cancer, PD-L1, programmed death ligand 1
PHACTR3: phosphatase and actin regulator 3
PLK1: polo-like kinase 1
PLXNA4: plexin A4
PKM2: pyruvate kinase isozyme M2
PML: promyelocytic leukemia
PRKN: parkin RBR E3 ubiquitin protein ligase
PTEN: phosphatase and tensin homolog
PTM: post-translational modifications
RASL10B: RAS-like family member 10B
RBPMS2: RNA-binding protein with multiple splicing 2
SERPINA5: serpin family A member 5
shRNA: short hairpin RNA
SNORD17: small nucleolar RNA, C/D box 17
SOX2: SRY-box transcription factor 2
SPC: side population stem-like cells, SSA/P, sessile serrated adenoma/polyp
STAC2: SH3 and cysteine-rich domain containing protein 2
STX19: syntaxin 19
SVM: support vector machine
SYTL4: synaptotagmin-like 4
S100A14: S100 calcium-binding protein A14
TCGA: The cancer genome atlas
TCIA: The cancer immunome atlas
TFF3: trefoil factor 3
TRIM25: tripartite motif containing 25
URGs: ubiquitination-related genes
VEGFR2: vascular endothelial growth factor receptor 2
ZMYND8: zinc finger MYND-type containing 8
