# Supplementary figures and images for: Identification of prognostic biomarkers and development of a prediction model for prostate cancer
Source: Front Immunol. 2026 Jan 5;16:1709264. doi: 10.3389/fimmu.2025.1709264 (PMC12813875; doi:10.3389/fimmu.2025.1709264)

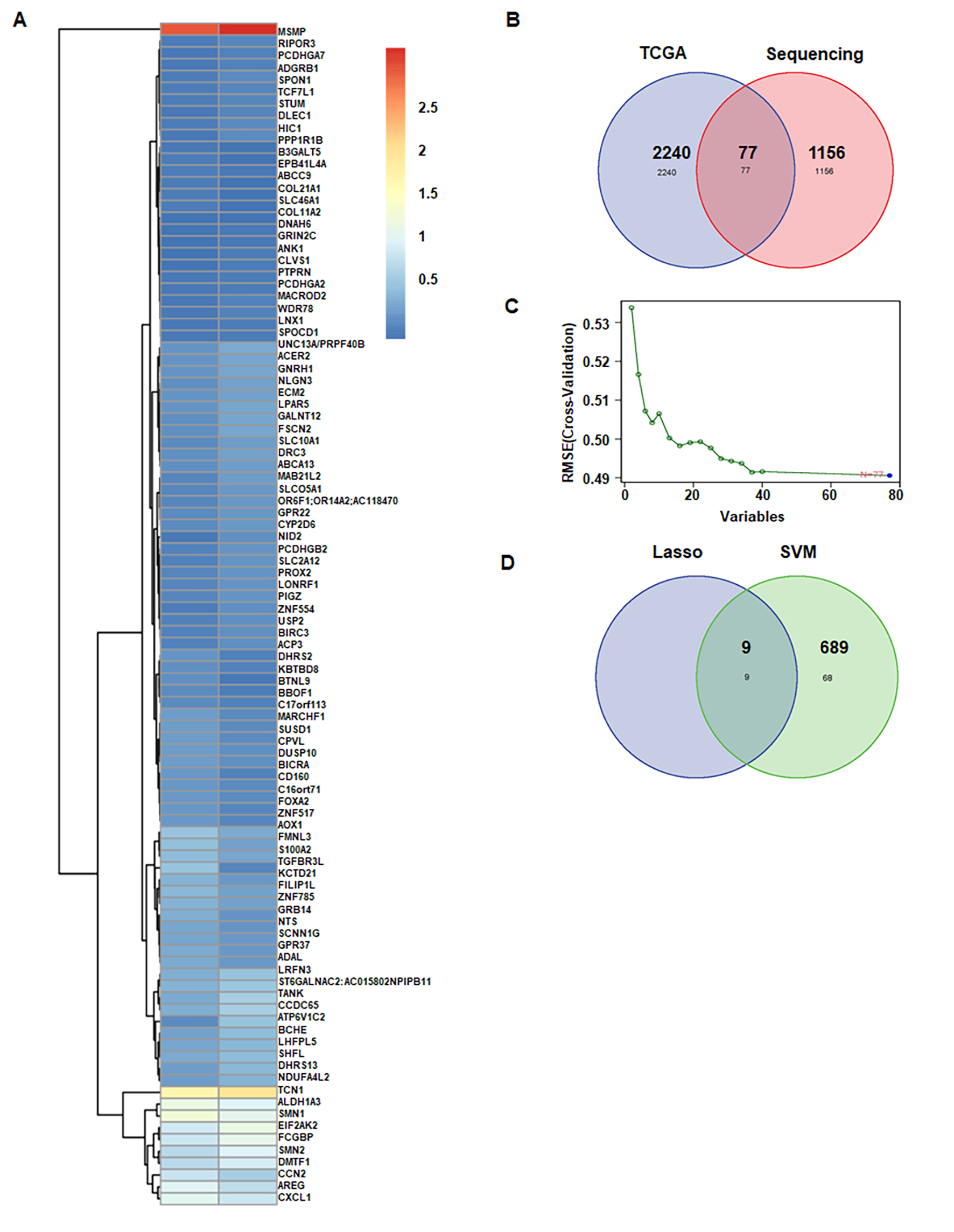

Supplement: Supplementary file 1 [file Image1.tif]

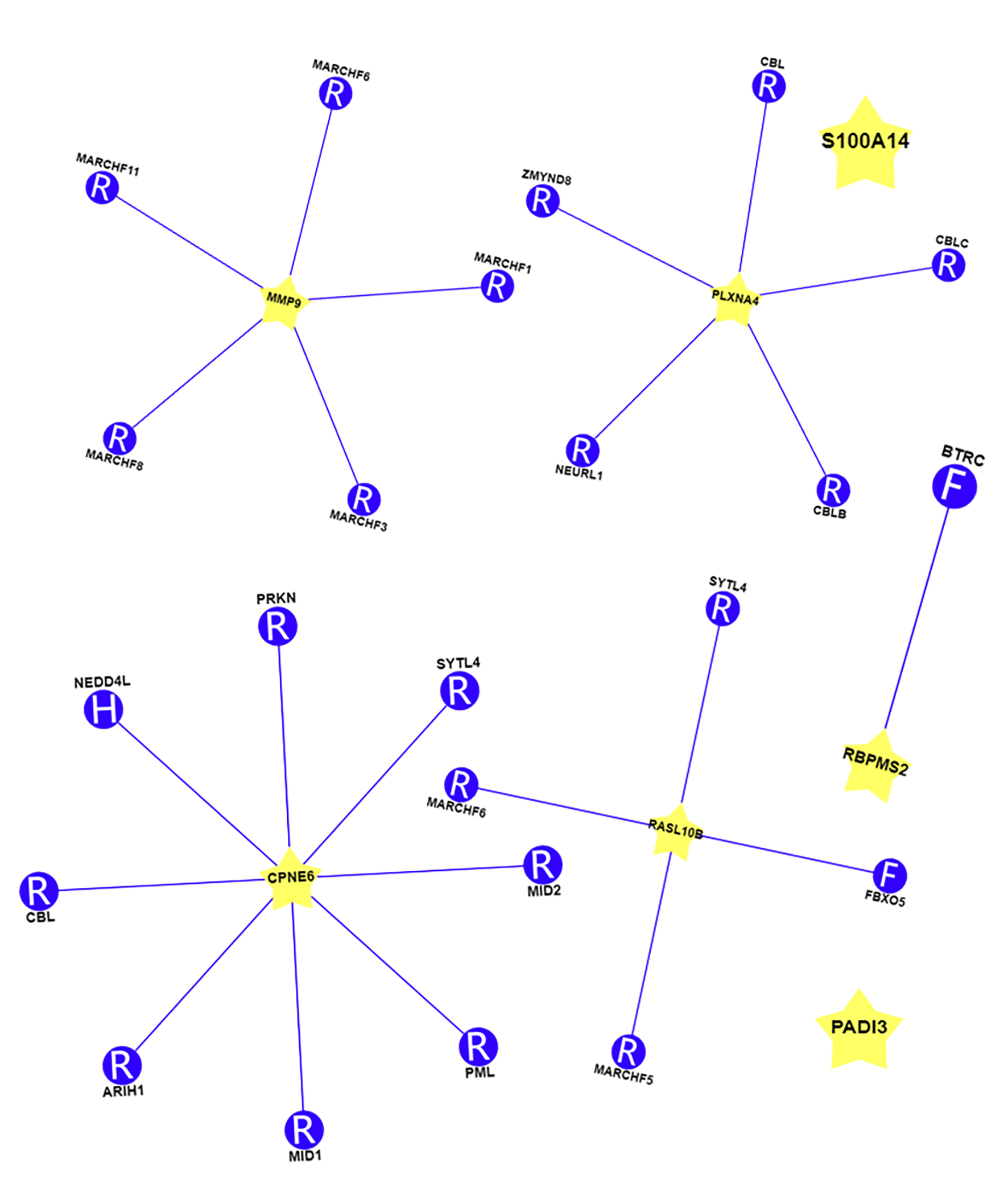

Supplement: Supplementary file 2 [file Image2.tif]

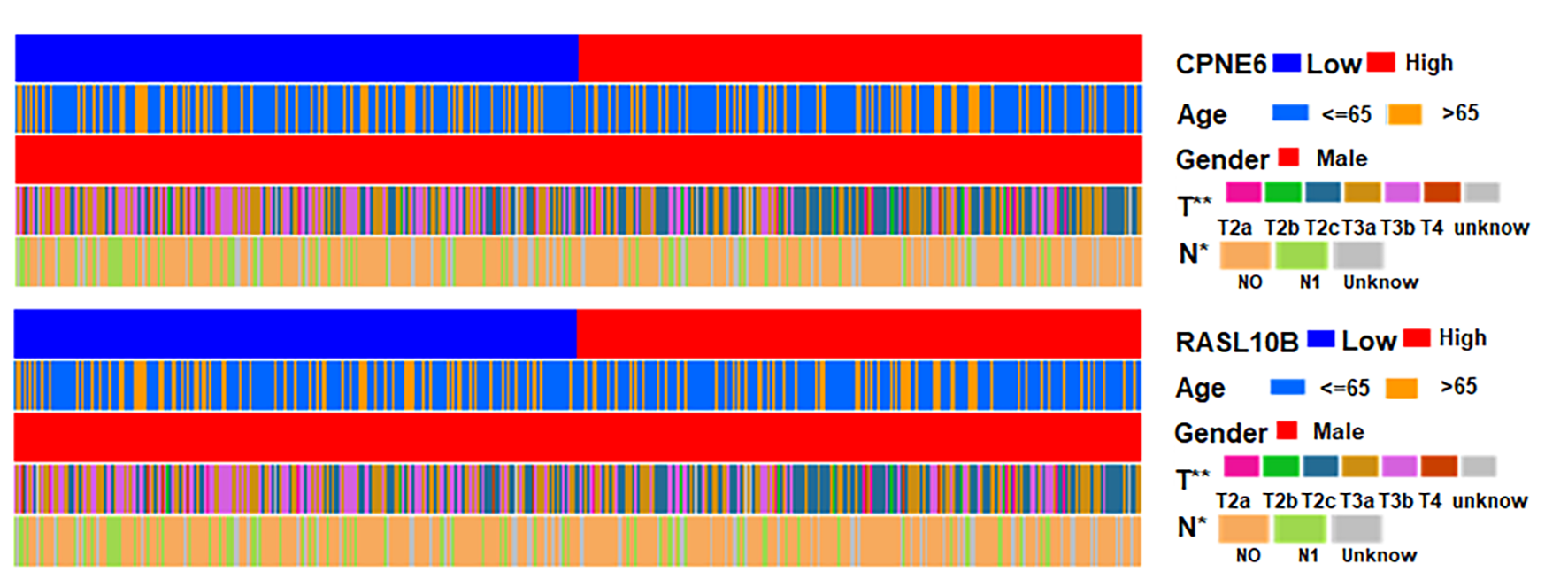

Supplement: Supplementary file 3 [file Image3.tif]

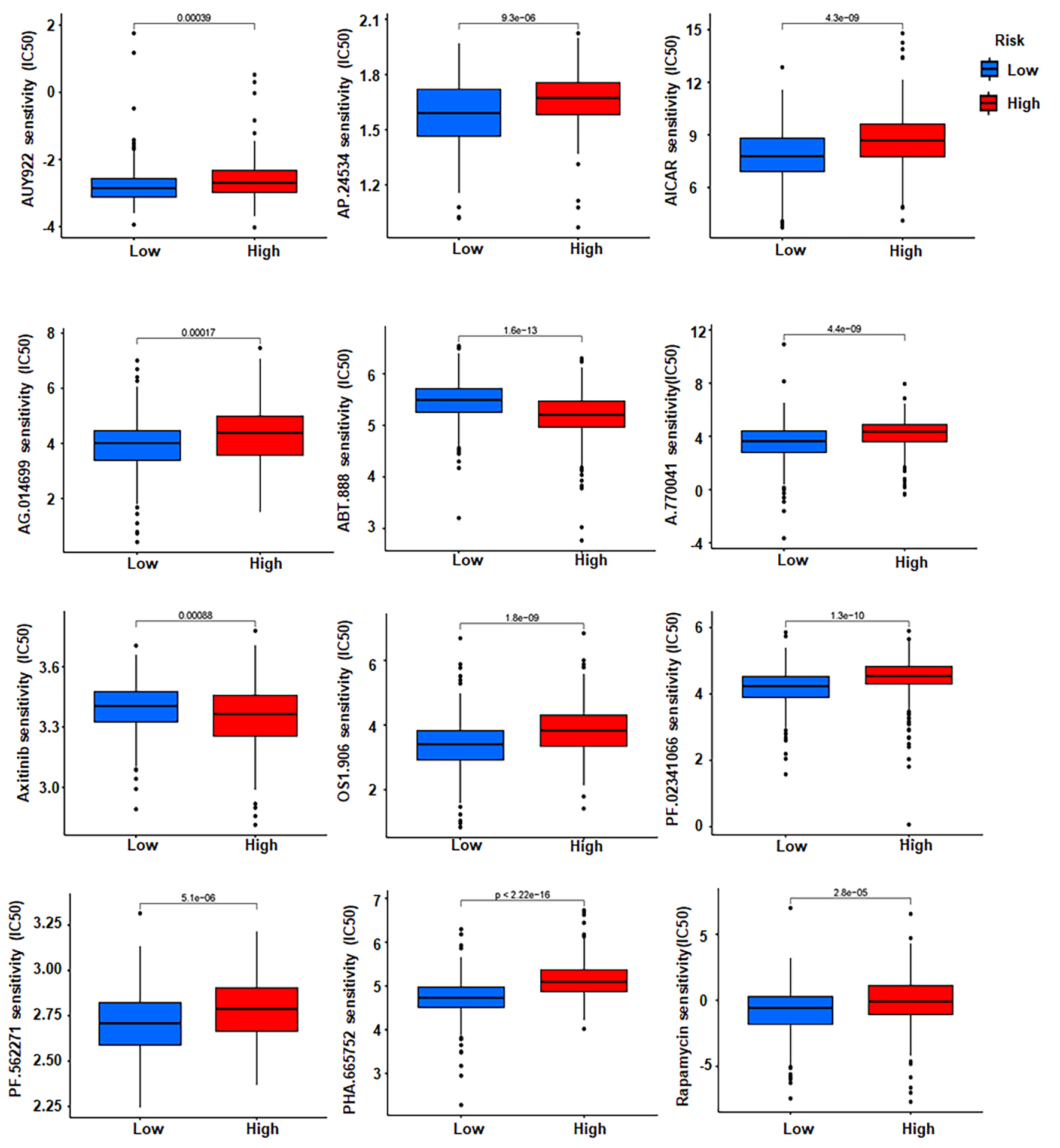

Supplement: Supplementary file 4 [file Image4.tif]

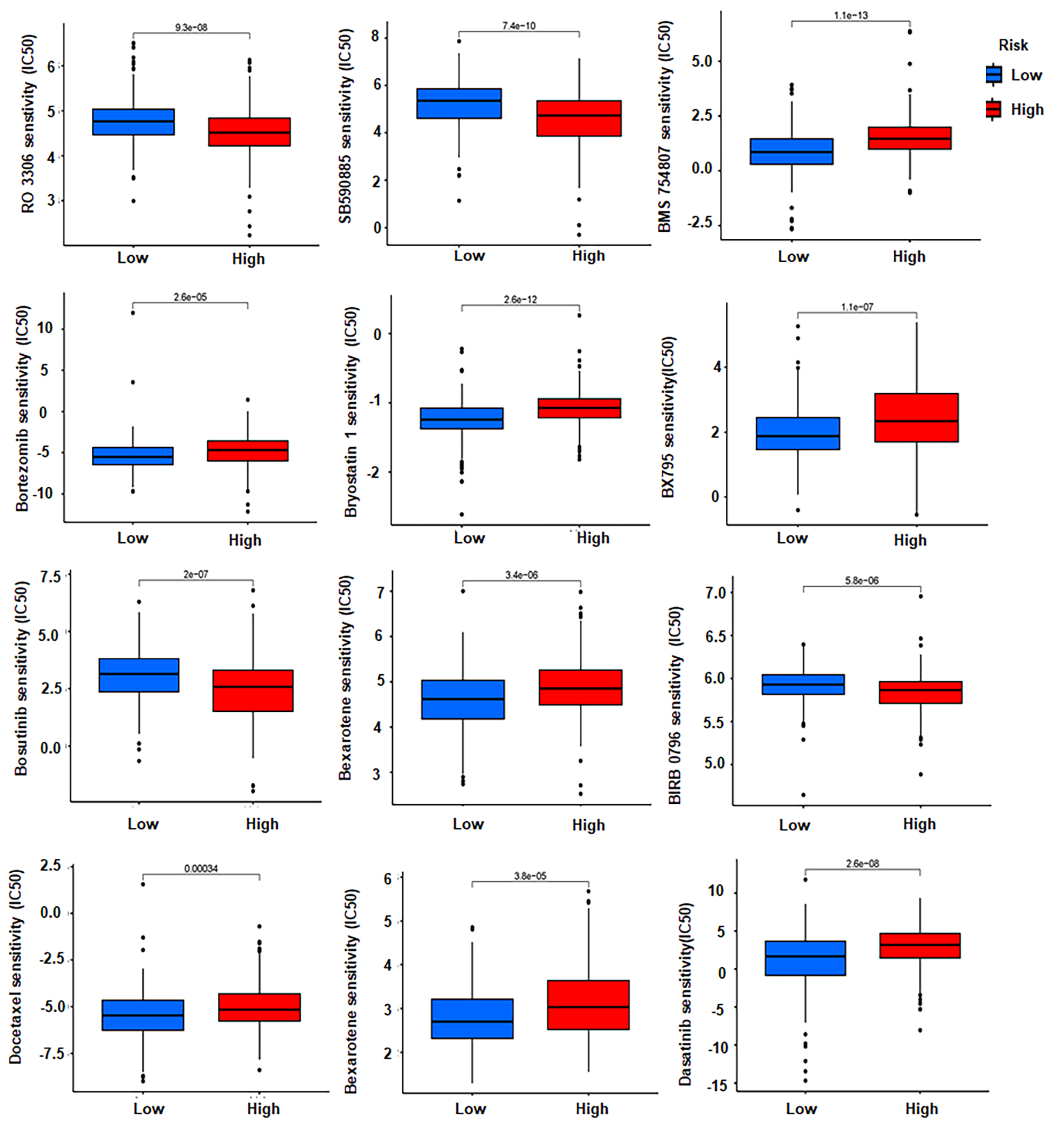

Supplement: Supplementary file 5 [file Image5.tif]
