# Supplementary material for: Identification of prognostic biomarkers and development of a prediction model for prostate cancer
Source: Front Immunol. 2026 Jan 5;16:1709264. doi: 10.3389/fimmu.2025.1709264 (PMC12813875; doi:10.3389/fimmu.2025.1709264)
Supplement: Supplementary file 6 [file DataSheet1.docx]

**Supplementary figure legends**

Supplementary figure 1: Identification of key genes.

(A) Barplot of gene expression profiles in PC3 and SPC Cells.

(B) Intersection of TCGA and sequencing data.

(C) Identification of Key Genes Using Support Vector Machine (SVM) Analysis.

(D) Venn Diagram of Seven Common Biomarkers Identified by Support Vector Machine and Lasso Methods. The chart displays the results of using support vector machine (SVM) and lasso methods to identify common biomarkers, represented in a Venn diagram.

Supplementary figure 2: Prediction network of ubiquitin ligase-substrate interactions of seven UR-BGs.

Supplementary figure 3: Clinical Feature Heatmap of CPNE6 and RASL10B. Rows represent clinical features, such as age, gender, cancer stage.

Supplementary figure 4: Drug sensitivity analysis. The box plots of the estimated IC50 for the most sensitive chemotherapeutic drugs.

Supplementary figure 5: Drug sensitivity analysis. The box plots of the estimated IC50 for the most sensitive chemotherapeutic drugs.
